# Therapeutic Potential of Engineered Virus-like Particles of Parvovirus B19

**DOI:** 10.3390/pathogens12081007

**Published:** 2023-08-02

**Authors:** Ignacio Sánchez-Moguel, Carmina Montiel, Ismael Bustos-Jaimes

**Affiliations:** 1 Departamento de Bioquímica, Facultad de Medicina, Universidad Nacional Autónoma de México (UNAM), Mexico City 04510, Mexico; moguelsig@gmail.com; 2 Departamento de Alimentos y Biotecnología, Facultad de Química, Universidad Nacional Autónoma de México (UNAM), Mexico City 04510, Mexico; carmina@unam.mx

**Keywords:** parvovirus-like particles, protein engineering, enzyme nanocarriers, nanobiotecnology, nanomedicine

## Abstract

Virus-like particles (VLPs) comprise one or many structural components of virions, except their genetic material. Thus, VLPs keep their structural properties of cellular recognition while being non-infectious. VLPs of Parvovirus B19 (B19V) can be produced by the heterologous expression of their structural proteins VP1 and VP2 in bacteria. These proteins are purified under denaturing conditions, refolded, and assembled into VLPs. Moreover, chimeric forms of VP2 have been constructed to harbor peptides or functional proteins on the surface of the particles without dropping their competence to form VLPs, serving as presenting nanoparticles. The in-vitro assembly approach offers exciting possibilities for the composition of VLPs, as more than one chimeric form of VP2 can be included in the assembly stage, producing multifunctional VLPs. Here, the heterologous expression and in-vitro assembly of B19V structural proteins and their chimeras are reviewed. Considerations for the engineering of the structural proteins of B19V are also discussed. Finally, the construction of multifunctional VLPs and their future potential as innovative medical tools are examined.

## 1. Introduction

Virus-like particles (VLPs) are composed of some or all of the structural components of viruses, excluding their genetic material. The lack of genetic material makes these particles non-infectious and safe for use. At the same time, they retain most or all of their structural properties, such as cell-tagging and antigenic properties [1]. VLPs can also be used to study virus–receptor interactions and the pathways virions use to internalize into host cells [2]. In addition, these particles can also be used directly as vaccines or as scaffolds for antigen presentation in the development of vaccines [3,4]. Currently, VLP-based vaccines are commercially available, including Gardasil and Cevarix for human papilloma virus (HPV), GenHevac B and Bio-Hep for hepatitis B Virus (HBV), Epaxal for hepatitis A Virus (HAV), Inflexal V for influenza virus, and Hecolin against hepatitis E virus (HEV) [5]. VLP-based vaccines against chikungunya virus, Ebola virus, influenza A virus, norovirus, Norwalk virus, respiratory syncytial virus, rotavirus, and SARS-CoV-2 are in development [5]. Theoretically, it is possible to produce VLPs from any virus, except for viruses whose assembly is based upon their genetic material. Therefore, several animal and plant viruses and bacteriophages have been used to produce VLPs for vaccination [6]. Moreover, the empty internal core of VLPs can enclose different cargo molecules, such as proteins, nucleic acids, drugs, or inorganic particles for hyperthermia applications [7]. VLPs loaded with any cargo molecule must be supported by the nature of the cargo, its cytotoxic effect, and the benefit/cost ratio. For example, using VLPs as vectors to direct chemotherapeutic drugs to a set of specific cells may help reduce the cytotoxic effect in healthy cells and reduce the total drug doses, as the therapeutic concentration does not need to be systemic. For example, VLPs of the physalis mottle virus (PhMV) loaded with a cisplatin prodrug, either attached to the internal or the external surface of the VLP, were able to carry this anticancer molecule into cancer cell lines with high efficiency [8]. PhMV particles were internalized by cancer cells and then disassembled to release the cargo based on pH changes, and they successfully inhibited the growth of xenograft breast tumors in mice.

Many studies have demonstrated that VLPs can be accumulated naturally in tumors because of their defective vascular structure [9,10]. Moreover, additional molecules can be included on the surfaces of VLPs to promote the internalization of these particles by including molecules as vitamins that the rapid-growing cancer cells highly demand [11] or by attaching peptides or proteins promoting specific cell internalization [12]. Noticeably, VLPs for biomedical applications must include properties that may be added via protein engineering or bioconjugation.

Here, the potential applications of parvovirus B19 (B19V) VLPs are discussed with an emphasis on protein engineering and the in vitro self-assembling advantages for developing potential therapeutic biocompatible materials. B19V is a human pathogen, and its potential application as an anti-B19V vaccine and for presenting heterologous epitopes is direct. In contrast, for applications outside the vaccine development field, being a human pathogen may be seen as a drawback. However, B19V offers a chance to gain access to its natural host cells and other non-permissive human cells. Moreover, any VLP can be hidden from the immune system by mutations in epitope regions or by covering it with stealth polymers [13,14]. Consequently, most of the weight for choosing a VLP platform relies on its ease of production and downstream manipulation, and B19V VLPs can be produced in bacteria, yeasts, insect cells, and cultured mammalian cells, and are stable enough for manipulation. The B19V capsid is neither glycosylated nor has disulfide bonds, so its expression in bacteria is limited only because capsid proteins accumulate as inclusion bodies (IBs) [15], a state that helps to obtain the proteins in high yields and purity levels for further applications [16].

## 2. B19V Structural Proteins and Virus-like Particles

B19V is an icosahedral virus whose capsid comprises 60 subunits of the major protein, VP2 (~95%), and the minor protein, VP1 (~5%). These proteins are encoded in its ~5.6 kb ssDNA genome in a single ORF with two start sites [17,18]. For this reason, VP1 and VP2 are identical, except for 227 additional residues in the N-terminal region of VP1, the so-called VP1 unique region (VP1u). This is common among parvovirus family members that have an ORF encoding for two or three viral particle (VP) proteins to assemble the viral capsid, and all VP proteins use the same termination codon [19].

VLPs of B19V have been constructed through the heterologous expression of VP2, VP2 + VP1, and VP1 proteins in different expression systems (Table 1). VP1 is a natural chimeric form of VP2 in B19V; therefore, this suggests that attaching heterologous protein domains at the N-terminus of VP2 is possible, producing VLPs with new functions.

**Figure 1:**
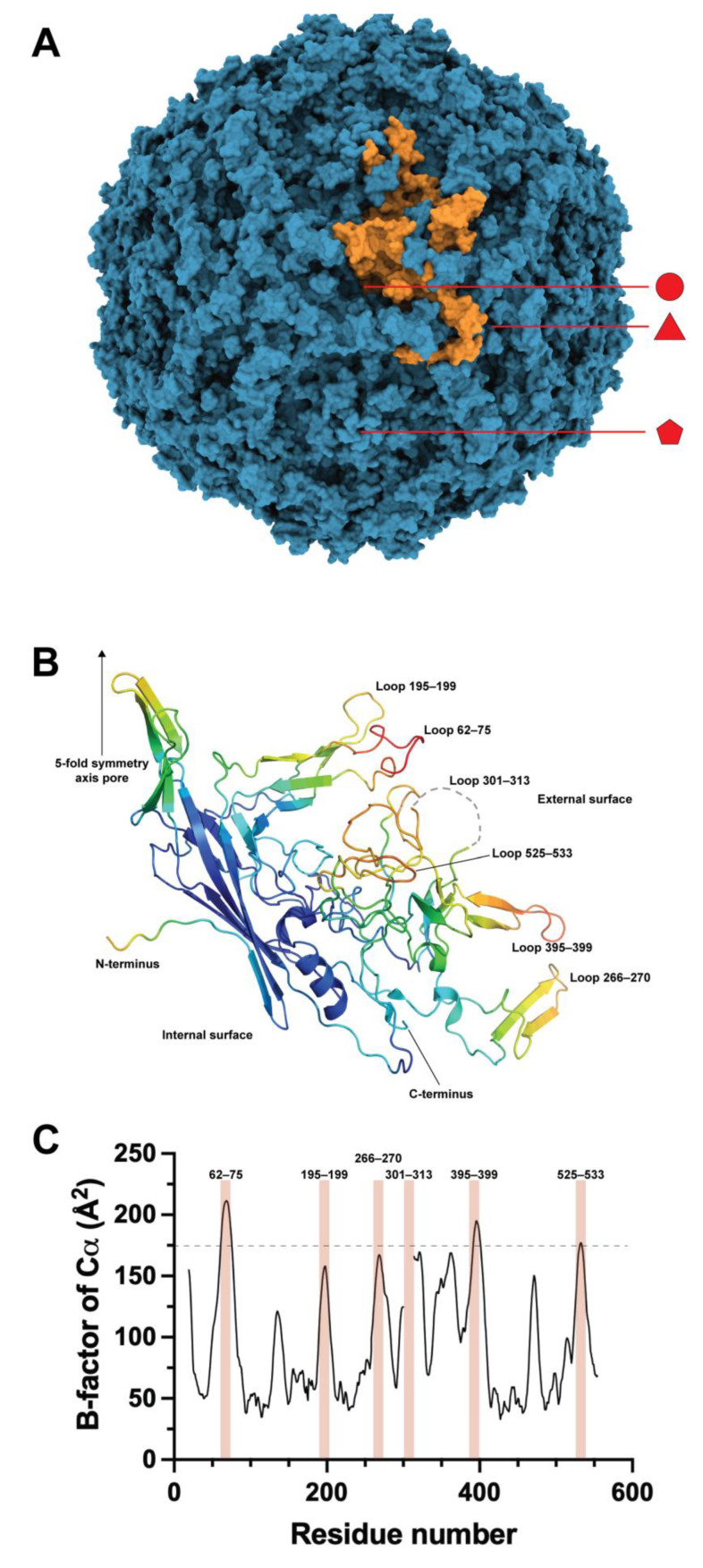
(**A**) Structure of the B19V VLP conformed by the VP2 protein (PDB 1S58). In orange is highlighted a monomer of the capsid. The 2-fold (circle), 3-fold (triangle), and 5-fold (pentamer) axes are indicated. (**B**) Structure of the monomer of VP2 in the capsid of B19V colorized by B-factors. The upper right side corresponds to the external surface of the VLPs and the bottom left side corresponds to the internal surface of the particle. The N-terminus corresponds to residue 19. (**C**) B-factor graph for the Cα of the B19V capsid. The most prominent values are highlighted with reddish bars and correspond to the external surface loops shown in panel B. The arbitrary limit of 175 Å^2^ in the B-factor for loop engineering is shown.

The structure of the B19V-VP2 VLPs was elucidated at a resolution of 3.5 Å via X-ray crystallography (Figure 1A) [26]. Most of the residues showed electron density, except for 18 N-terminal residues and 13 residues of the surface loop 301–313. This loop is of interest for engineering purposes as its high mobility suggests that it has no structural compromise, meaning it can be modified. Loop engineering is a common approach for inserting functional peptides or protein domains in proteins and parvovirus structural proteins, such as in the canine parvovirus [27], and also in B19V VLPs by our group [28,29] and recently by the group of Morita [30].

The core of the B19V VP2 structure consists of an eight-stranded, antiparallel β-barrel consisting of two β-sheets, and the rest of the structure comprises insertions connecting the β-barrel strands. The antiparallel β-barrel is a common feature of mammalian parvoviruses and other icosahedral viruses, and the loops have different lengths and aminoacidic compositions but have homologous positions among parvoviruses [31]. The capsid appears to be stabilized by interlocking neighboring VP2 molecules through surface loops. However, some surface loops do not participate in intermolecular contacts; thus, they are also candidates for protein engineering. Finally, the final four amino acids of the C-terminal of VP2 are buried within the capsid, pointing to the internal capsid core, making this site an attractive place to incorporate molecules that are supposed to be enclosed in the protein shell. Regrettably, no examples of functional molecules at the C-terminus are yet available. However, VP2 and most of its chimeras expressed in *E. coli* have a C-terminal 6xHisTag for IMAC purification [15]. In contrast, the observed N-terminus of the VP2 structure is located on the inside of the capsid, close to a fivefold axis. The 18 residues not constructed in the structural model are disordered, so this part of the structure is not critical for the capsid assembly.

B19V wildtype virions carry about 5% VP1, and CryoEM analyses of these capsids show that the N-termini of VP2 are projected to the outer side of the capsid through the 5-fold axis pore, allegedly because of the crowded interior of the capsid. VLPs, however, have no enclosed genomes; thus, the reduced steric restrictions at the internal core of the capsid may hold the VP2 protein N-termini inside the capsid [32]. It has also been reported that VP1u is located on the external surfaces of the B19V virions [33], while another group found that not all of VP1u is accessible on the surface of the virion and that pH or temperature changes stimulate conformational changes in the capsid that cause the entire region to be exposed [34]. Considering that VP1 occurs in 5% of the whole capsid, this is 3 out of the 60 subunits of the complex. Even with this low density, some of its residues are exposed on the surface, and it is plausible that some N-terminal extensions of VP2 can also be exposed on the surfaces of the VLPs constructed entirely from these chimeras. Several groups later demonstrated this principle by engineering the N-terminal region of VP2/VP1.

VP1u has been studied, and some of its roles in infection have been described, including the presence of a receptor-binding domain for cell internalization and a phospholipase-A2 (PLA2) domain for lysosomal escape [35,36,37,38]. It has been reported that VP2 can produce VLPs by itself, while VP1 alone barely produces VLPs via heterologous expression in insect cells. In addition, truncated forms of VP1 gain competence for self-assembly as VP1u is shortened [39]. This result suggests a limited tolerance to N-terminal extensions in VP2, both in size and in the number of subunits per capsid. Therefore, it is reasonable that other N-terminal peptides or even small protein domains may be well tolerated in that position, either alone or coassembled with VP2.

## 3. Engineering of the N-Terminal Region of VP2/VP1

Several groups have taken advantage of the presence of the N-termini of VP2/VP1 on the external surface of the B19V capsid, adding heterologous peptides to the N-termini of VP2 to use the resulting VLPs as a scaffold for antigen presentation, including antigenic determinants of dengue 2 virus (DEN-2), Bacillus anthracis, herpes simplex virus 1 (HSV-1), mouse hepatitis virus (MHV), and the syncytial respiratory virus (SRV) [40,41,42,43]. Table 2 summarizes the results of these investigations, suggesting that the fusion of antigens to the N-terminus of VP2 has a large chance to succeed in producing neutralizing antibodies.

Nevertheless, antigen presentation is not the only role these particles can play. Our group has included functional peptides and proteins fused or conjugated at the N-terminus of VP2. For example, the bioconjugating peptide SpyTag was genetically fused at the N-terminus of VP2, and the expressed protein was assembled into VLPs displaying this peptide on their external surface. Those SpyTag-VLPs were successfully decorated with an α-glucosidase from *S. cerevisiae* previously fused with the bio-orthogonal partner of the peptide SpyTag, the SpyCatcher domain [44]. The SpyTag/SpyCatcher system and its derivatives are well-established protein bioconjugation technologies [45]. The kinetic and stability properties of the conjugated enzyme were studied. The thermal stability of the α-glucosidase activity was minimally increased at pH 5 and 37 °C, changing the half-life (t_1/2_) of 40 min for the unconjugated enzyme to 48 min for the VLP-bound α-glucosidase. Additionally, a three-fold increase in the *k*_cat_ of the coupled enzyme was observed without changes in *K*_M_. The resulting particles, carrying the α-glucosidase activity, also showed activity on glycogen so that they could be used for the delivery of this activity into the lysosomes of people suffering from glycogen storage disease type II (Pompe disease) caused by a deficiency of α-1,4-glucosidase in lysosomes as a form of enzyme replacement therapy [46].

A second example of functional peptide addition at the N-terminus of VP2 is the peptide PreS_21–47_, derived from the hepatitis B virus (HBV) [47]. This peptide is necessary to target HBV to hepatocytes [48]. The sodium taurocholate cotransporting polypeptide (NTCP) has been described as the receptor of HBV by direct interactions with PreS_21–47_ [49], and then the internalization of HBV is mediated by clathrin-dependent endocytosis [50]. Therefore, VLPs constructed with the peptide PreS_21–47_ fused to VP2 efficiently targeted the chimeric particles to HepG2 cells. Remarkably, in this experiment, VLPs were constructed with a second chimeric form of VP2, the SpyTag-VP2 mentioned above. The hybrid particles, decorated with both peptides, PreS_21–47_ and SpyTag, were decorated with a GFP protein fused to the SpyCatcher domain. As a result, the VLPs harboring the PreS_21–47_ peptide were also fluorescent, and the efficient internalization of the particles into hepatocytes was quickly confirmed by confocal microscopy.

Another special feature was included in the construction of the hybrid PreS_21–47_-VP2 and SpyTag-VP2 particles. While the hybrid VP1/VP2 particles produced in eucaryotic cells were carried out by the coexpression of VP1 and VP2 genes in the same cell (Table 1), the hybrid particles produced with VP2 chimeras expressed in bacteria were manufactured by mixing the denatured chimeras and then set to refold and self-assemble via dialysis. VP2 and its chimeras are expressed in *E. coli* in the form of inclusion bodies (IBs) that can be resuspended in guanidine hydrochloride (GuHCl) and then purified by IMAC, given that VP2 and its chimeras have a C-terminal 6x-HisTag that does not impair its competence for self-assembly [15,51]. VP2 and its chimeras are purified by IMAC before self-assembly, given that the C-terminus with the 6x-HisTag would not be accessible to the surface of the assembled nanoparticle. This method for hybrid particle assembly opens the door to constructing chimeras harboring a large variety of peptides. Taking into consideration that 60 subunits of VP2 make up these particles and that the assembly is a statistical event in which theoretically bias is not present for the incorporation of one chimera over others, there is a high probability of having all of the chimeras combined in a single particle when a small number of chimeras is used in the coassembly. Moreover, as a proof of concept, our group loaded small fragments of dsDNA into the particles by adding them in the dialysis step of refolding and assembly [52]. This strategy can be optimized for selected small RNAs, bioactive peptides, or small drugs requiring a tagging vector for a better therapeutic output.

In addition to functional peptides, large domains could be added at the N-terminal of VP2. In a pioneering study by Miyamura and coworkers [53], it was demonstrated that an enzyme can be present on the surface of a B19V VLP. For this purpose, a sequence encoding the 147 residues of hen egg white lysozyme (HEL) was used to substitute the VP1u, either partially or entirely, and then coexpressed with VP2 in Sf cells. The resulting VLPs presented HEL domains on their external surfaces, as demonstrated by the immunoprecipitation, ELISA, and transmission electron microscopy of gold-labeled anti-HEL antibodies. The HEL-VLPs showed activity towards the *Micrococcus luteus* cell wall, demonstrating the potential of B19V VLPs as a scaffold for protein presentation and as enzyme carriers. However, the authors found that the range of HEL domains is low, at between 0.5 and 5 per capsid, while the expected range was between 24 and 30 because of the transforming methods used on Sf cells. It was suggested by the authors that this is because most HEL domains remain inside the capsid.

Remarkably, in the in vitro refolding–assembly method, not every single N-terminal chimera of VP2 can form VLPs. N-terminal chimeras of VP2 with the human dihydrofolate reductase (DHFR), α-glucosidase from *S. cerevisiae* (Ima1p), superfolder GFP (sfGFP), and *Bacillus pumilus* lipase A (BplA) were constructed and expressed (unpublished data). None of them could efficiently self-assemble into VLPs, suggesting that large heterologous N-terminal domains impose steric constraints on the association of proteins to form VLPs. Here, the coassembly of these particles with 75% of VP2 allowed the formation of VLPs for all N-terminal chimeras. Regrettably, only the hybrid VLPs with the heterologous domains of sfGFP and BplA showed fluorescence and lipase–esterase activity, respectively. On the other hand, the DHFR and Ima1p domains showed no significant activity. Interestingly, VP2 and the VP2 N-terminal chimeras of sfGFP and BplA were successfully coassembled to produce three-component hybrid VLPs, simultaneously displaying fluorescence and lipase–esterase activity.

Recently [54], multiepitopic peptides were also fused to the N-termini of VP2, the neoepitopes Tmtc2, Gprc5a, and Qars, identified by next-generation sequencing of the 4T1 cell line as epitopes recognized by CD8 cells [55] and the survivin peptide 66–74, a tumor-associated antigen [56]. The four peptides were arranged in a single genetic construction fused to the N-terminus of VP2, then expressed in *E. coli* as IBs. The recombinant chimera was purified under denaturing conditions and then refolded and self-assembled into VLPs. These particles were administered to mice in which 4T1 cells were previously implanted to establish tumors. The 4T1 cell line is a mammary carcinoma model, which is highly tumorigenic and invasive and spontaneously metastasizes from the mammary gland to other tissues, including the lungs and liver [57]. As foreseen, VLPs carrying the multiepitopic peptide significantly delayed tumor growth. Surprisingly, the control VLPs made only with VP2 produced a minor but significant delay in tumor growth, an intriguing result that deserves further research. Moreover, multiepitope-carrying VLPs also led to reduced lung macro-metastasis of 4T1 cells and lung tumor growth. Finally, treatments with the chimeric VLPs induced specific proliferative responses of CD8 and CD4 T lymphocytes, as well as granzyme-B production in lymphatic nodes close to the tumor. This is an encouraging result, as mammary carcinoma is one of the most lethal forms of cancer [58].

In a different approach to fighting cancer, breast-cancer-related epitopes targeting the insulin-like growth factor receptor (IGF-1R) were used to construct chimeric VLPs [59]. IGF-1R is overexpressed in about 50% of breast cancers and plays a central role in cell growth, metastasis, and angiogenesis [60]. Peptides P8 [61] and 249 [62], predicted as epitopes of the extracellular domain of IGF-1R, were genetically fused to the N-terminus of VP2. The chimeras of VP2 were expressed as IBs, purified, and used for the manufacture of VLPs composed of 249-VP2 or P8-VP2, or a hybrid made up of 249-VP2 and P8-VP2. These particles, applied as a prophylactic, protected against tumor formation and the growth of 4T1 cells inoculated in mice. This preliminary study encourages a translational study for the application of cancer vaccines in humans.

Considering that the capsid of natural virions is also a hybrid particle composed of VP1 and VP2, our group applied the in vitro coassembly method to construct VP1/VP2 VLPs [23]. Our results showed that any ratio of VP1/VP2 produced VLPs, and surprisingly VP1 alone was also able to self-assemble into VLPs. The correct folding of VP1u was studied through its functional properties, i.e., the presence of PLA2 activity and its ability to tag cells displaying integrin α5β1, such as HepG2 cells, a validated non-permissive model for B19V infection [63]. Our results showed that hybrid VP1/VP2 or VP1-only VLPs have PLA2 activity and can also internalize into HepG2 cells through their endosomal system. Previous studies demonstrated that it was possible to produce hybrid VLPs harboring different ratios of VP1/VP2, at up to 41% of VP1, by coinfecting insect cells with different ratios of the multiplicity of infection (MOI) of the baculovirus carrying either VP1 or VP2 genes [64]. While this is an interesting approach for increasing the amount of VP1, it is unclear if this is the maximum level of VP1 that VLPs can harbor in insect cell systems. Recent studies showed that the coexpression of VP1 and VP2 can produce VLPs with up to 25% VP1 in the yeast *Hansenula polymorpha* by using strong promoters and terminator sequences [24]. Finally, the expression of VP1/VP2 VLPs in human 293 T cells was achieved by transforming the cells with equimolar plasmid doses, obtaining VLPs harboring a VP1/VP2 ratio close to one [25].

## 4. VP2 Loop Engineering

A second approach for VLP engineering was inserting heterologous sequences in the external surface loops. The pioneering work on loop modifications was carried out in 1994 [41], before the existence of the crystallographic structure of the B19V capsid. The insertion of linear epitopes of HSV and MVH was then carried out in a region of VP2 predicted as a loop by bioinformatics, between residues 274 and 275, a region that in the crystallographic structure lies in the stem of a loop, in a region forming a two-strand β-sheet and a few residues away from the actual tip of the loop (residues 265–271) (Figure 1B). Nevertheless, epitope insertions in this site did not impair the chimeric VP2 from producing VLPs. However, when used as antigens to immunize mice, these particles produced lower antibody titers against HSV and MVH than VLPs carrying the same insertions at the N-termini of VP2. This loop engineering was the first demonstration of the structural plasticity of the VP2 core away from the N-terminus.

With the elucidation of the structure of the capsid of B19V, a vast amount of information became available to resume the engineering of surface loops of B19V VLPs. First, it became possible to define the location and size of the loops, as depicted in Figure 1B. The analysis of the crystallographic B-factors for the C*α* atoms of the structure gave insight into the loops with the largest mobilities that can be correlated with a less compromised structure. As observed in Figure 1C, the highest B-factor values correlate well with the surface loops of the crystallographic structure. In order to reduce the number of engineering candidate loops, our group set an arbitrary limit of 175 Å^2^, which was only exceeded by three loops, 62–75, 395–399, and barely 525–533. In addition, loop 301–313, which showed no electron density in the crystallographic structure due to its high mobility, was also considered among the engineering candidates.

Loop 62–75 was engineered to harbor a 64-residue peptide from the F protein of the RSV [28]. The engineered protein produced VLPs by the refolding–assembly method, either alone or coassembled with VP2.

While loop 301–313 was also successfully engineered to present the same 64-residue peptide of the F protein of the RSV (unpublished data), a more ambitious goal was set for engineering this loop—to display a complete and functional protein on the surface of the B19V VLPs [29]. This procedure supposed a double risk—the first being that the inserted protein could obstruct the correct folding of the VP2 domain split by the hosted protein domain, and the second being the opposite, the incorrect folding of the hosted domain due to interferences produced by the flanking VP2 segments. Our first attempt was the introduction of an EGFP domain (238 aa) between residues 307 and 308 of VP2 by genetic fusion, adding only a Gly residue at each side of the hosted protein to provide additional structural freedom to the folding process, considering that the 13-residue loop is highly mobile. The protein was expressed in *E. coli* as IBs, which were processed as the VP2 IBs and then refolded to produce VLPs. The resulting particles were in the diameter range of VLPs, and regrettably were not fluorescent. In contrast, the lipase–esterase BplA (181 aa), inserted in the same position and flanked again by single Gly residues, produced particles with diameters in the range of VLPs and had lipase–esterase activity. The lipase immobilized on the surface of the VLPs showed a 20-fold decrease in specific activity, which may be related to rigidity produced by its attachment to the surface of the particles, reducing the thermal fluctuations required by enzymes to work properly. On the other hand, the first-order thermal inactivation constant of the immobilized lipase was reduced 7-fold, producing a longer half-life to the immobilized enzyme at 40 °C, from 0.14 to 1.01 h. Taking into consideration the possible effects of the reduced freedom degrees imposed by the attaching of the N- and C-termini of the hosted protein to the surface of the VLP, we explored the effect of a longer linker and a fast-folding domain, the sfGFP (238 aa); this protein is a derivative of the EGFP with mutations that enhance its folding rate [65]. This protein, and the addition of 11-residue flanking linkers (GGSGGSGGSGG), produced fluorescent IBs that were resuspended and then refolded to assemble VLPs. The produced particles were fluorescent, although their specific fluorescence intensity was about 5-fold lower than the free sfGFP. The stability of the immobilized sfGFP was assayed; however, the VLPs aggregated before fluorescence intensity was affected by temperature. Therefore, the selection of the protein to be hosted by the particles is critical, as well as the selection of linkers flanking it. Then, a preliminary study of the self-folding faculty of the enzyme to be hosted is strongly advised.

Loop 266–270 was also engineered to host the previously mentioned 64-residue peptide from the F protein of the RSV (unpublished data), confirming that this stem loop (262–275) can host heterologous peptides, as found by Brown and colleagues in 1994. Other loops have yet to be tested. However, the proven properties of loops 62–75, 301–313, and 266–270 are appealing enough to insert functional peptides or proteins to provide new functions to the VLPs.

## 5. VLP Vaccines against B19V

B19V has been identified as the causal agent of several diseases in humans, including erythema infectiosum in children, aplastic crisis in sickle cell disease patients, persistent anemia in immunocompromised patients, polyarthralgias or polyarthritis, and hydrops fetalis in pregnant women [66,67]. Infection by B19V usually produces the destruction of erythroid precursors in the bone marrow. In a few cases, pancytopenia, neutropenia, thrombocytopenia, hemophagocytosis, myocarditis, or hepatitis has been observed. In healthy individuals, the effect of a B19V infection may be asymptomatic. However, in anemic patients, the decline in red blood cells may lead to acute transient aplastic crises. Therefore, a vaccine against B19V has been envisaged for a long time. In 1991, it was shown that VP1/VP2 VLPs produced in insect cells were similar in antigenicity and immunogenicity to natural virions of B19V [68]. The first VLP-based vaccine candidate against B19V was reported 30 years ago by constructing hybrid VP1/VP2 VLPs and inoculating mice, guinea pigs, and rabbits [64]. In this research, different MOI ratios of the baculovirus carrying VP1 or VP2 were employed to infect *Spodoptera frugiperda* Sf-9 insect cells to obtain different ratios of VP1 and VP2 in the produced VLPs, and it was found that the higher the VP1/VP2 ratio, the higher the titer of neutralizing antibodies produced. This approach was later scaled to a bioreactor by considering several process variables, such as the temperature, harvest time, lactate concentration of dissolved O_2_, cell density, and MOI [69]. This study is relevant as viral infection is a statistical process, and not every cell receives the same number of infection events. Therefore, the expression of the viral proteins may be homogeneous in the sample but only some cells produce such a composition of viral proteins. By 2003, the first randomized, double-blind, phase 1 trial was carried out by inoculating 2.5 or 25 μg of the VP1/VP2 capsids over a ratio range of 25–75% with the adjuvant MF59C.1, an oil-in-water emulsion of squalene in citrate buffer [70]. All volunteers in this trial developed neutralizing antibodies that lasted at least one year, suggesting that this vaccine was safe and immunogenic. However, when this study was moved to a larger cohort of volunteers, three cutaneous manifestations appeared and the study was halted [71]. The authors argue that the PLA2 activity of the VP1u of VP1 may be responsible for such reactogenicity, as it could release precursors of potent inflammatory mediators. A second possibility the authors raised is that traces of baculovirus or insect cell proteins triggered the allergic reactions. Regrettably, the trial was not resumed even though the symptoms disappeared three days after their apparition. Later, in 2013, VP1/VP2 VLPs were constructed in *S. cerevisiae* instead of insect cells, and VP1 was mutated to remove its PLA2 activity, addressing the two main possible causes for the reactogenicity found in the previous trial [72]. The particles produced in *S. cerevisiae* were homogeneous in size and elicited a potent neutralizing response in mice. Similar VLPs were produced recently in *H. polymorpha*, lacking PLA2 activity and showing excellent preclinical properties [24]. In-vitro-assembled B19V VLPs composed with a desired VP1/VP2 ratio and carrying the correct mutations to PLA2 inactivation could also be attractive vaccine candidates. These proteins are produced in *E. coli* with high yields and are purified under denaturing conditions, removing most contaminants that may produce side effects in trials. To our knowledge, no currently active trials for B19V vaccines are in progress.

## 6. Conclusions and Expectations

**Figure 2:**
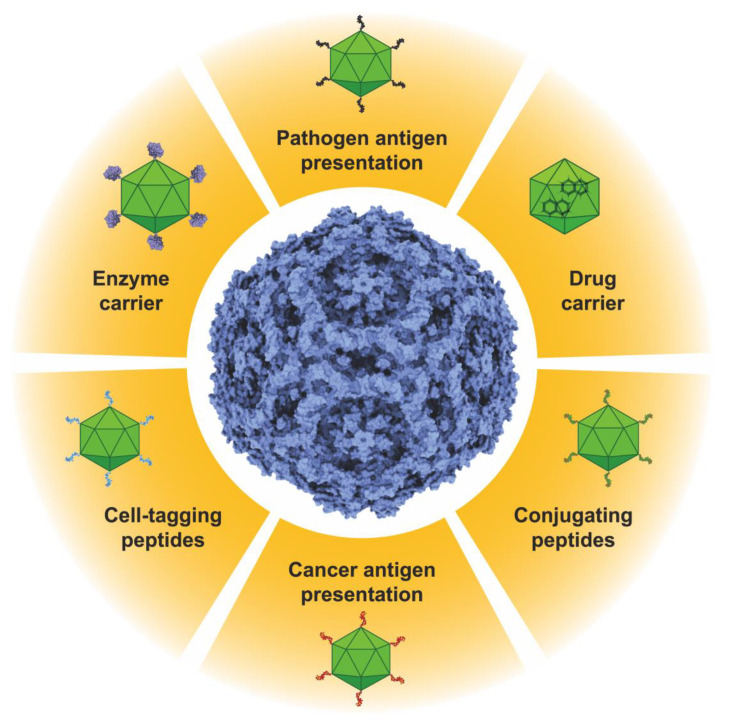
Potential applications of the B19V VLPs. It is possible to present antigens of transmissible or non-transmissible diseases on the surfaces of the particles. VLPs can be loaded with drugs, peptides, proteins, or nucleic acids. Enzymes or proteins can also be presented or immobilized on the surfaces of the VLPs. Functional peptides for cell tagging or for bioconjugation purposes can also be displayed on the surfaces of B19V VLPs. Bioconjugating peptides allow for these nanoparticles to be decorated with a wide variety of molecules with biotechnological applications. Two or more of these properties can be combined in a single particle, producing polyfunctional VLPs.

B19V VLPs are an exciting platform for potential biomedical applications (Figure 2). As reviewed here, they can present immunogenic epitopes for vaccine production or peptides for bio-orthogonal conjugation, allowing the decoration of these particles with a wide variety of proteins or peptides for any imaginable function. As stated above, they could be used as enzyme nanocarriers to treat lysosomal diseases or other metabolic diseases that can be alleviated by enzyme delivery. Additionally, these particles can carry more than one function by coassembly with other VP2-chimeras, so they can also be decorated with molecules for selective cell tagging, concentrating their therapeutic functions in a selected group of cells. In addition, the empty internal core of the B19V VLPs can be loaded with different molecules displaying therapeutic potential. Finally, with an adequate strategy, it could be possible to manufacture hybrid VLPs displaying peptides or proteins to direct the particles to specific tissues or cells and have specific duties. Simultaneously, these particles can be loaded with adequate bioactive molecules for synergic therapy.

B19V structural proteins can be produced in different heterologous expression systems, from bacteria and yeasts to insect and mammal cells. This flexibility allows for producing VLPs to display different peptides or proteins on the external surfaces of the nanoparticles, including those that may require post-translational modifications. B19V VP2/VP1 VLPs can be modified, either in the N-terminus or in surface loops, and such modifications are not mutually exclusive, thereby allowing them to harbor more than one new property in each subunit. Moreover, the possibility to regulate the ratio of VP2 and its chimeras by mixing different amounts of each protein during the folding–assembly process exponentially permits the accumulation of new properties in the built VLPs. This feasibility to gather properties is rare in other VLPs and promises to be the basis for new tools in nanomedicine.

## Figures and Tables

**Table 1:** Heterologous systems for the production of parvovirus B19 structural proteins and virus-like particles.

Proteins	Expression System	Reference
VP1 + VP2	Chinese Hamster Ovary cells	[20]
VP2 VP2 + VP1	*Spodoptera frugiperda* Sf cells	[21]
VP2	*Saccharomyces cerevisiae*	[22]
VP2	*Escherichia coli*	[15]
VP1 VP1 + VP2	*Escherichia coli*	[23]
VP1 + VP2	*Hansenula polymorpha*	[24]
VP1 + VP2	Human 293 T cells	[25]

**Table 2:** Presentation of heterologous antigens from infectious pathogens on B19V VLPs via fusion with the N-terminus of VP2/VP1 proteins.

Pathogen	Antigen Type	Antigen Length	Neutralizing Antibodies	Reference
DEN-2	E-glycoprotein domain III and fragments of this domain	12–100 aa	Yes, titer depends on the selected fragment	[40]
*B. anthracis*	Fragments of the PA subunit of the anthrax toxin	27–173 aa	Yes, titer depends on the selected fragment	[43]
HSV-1	Glycoprotein gD, epitope VII, residues 9–21	13 aa	Yes	[41]
MHV	Spike protein, Site A epitope	12 aa	Yes	[41]
SRV	Fusion glycoprotein F, antigenic site II, antigenic sites IV-VI	63 aa site II 57 aa sites IV-VI	N.D.	[42]

N.D. Not determined.

## Data Availability

Not applicable.
